# Cold Acclimation of *Trogoderma granarium* Everts Is Tightly Linked to Regulation of Enzyme Activity, Energy Content, and Ion Concentration

**DOI:** 10.3389/fphys.2018.01427

**Published:** 2018-10-30

**Authors:** Mozhgan Mohammadzadeh, Hamzeh Izadi

**Affiliations:** Department of Plant Protection, Faculty of Agriculture, Vali-e-Asr University of Rafsanjan, Rafsanjan, Iran

**Keywords:** cold-acclimated, fluctuating-acclimated, rapid cold-hardened, enzyme activity, ion concentration, Khapra beetle

## Abstract

In this study, cold hardiness and some physiological characteristics of the Khapra beetle, *Trogoderma granarium* Everts (Coleoptera: Dermestidae) larvae, were investigated under different thermal regimes, i.e., control, cold-acclimated (CA), fluctuating-acclimated (FA), and rapid cold-hardened (RCH). In all the regimes, the larval survival rate decreased with a decrease in temperature. CA larvae showed the highest cold hardiness following 24 h exposure at -15 and -20°C. Control larvae had the highest glycogen content (34.4 ± 2.3 μg/dry weight). In contrast, CA larvae had the lowest glycogen content (23.0 ± 1.6 μg/dry weight). Change in trehalose content was reversely proportional to changes in glycogen content. The highest myo-inositol and glucose contents were detected in CA larvae (10.7 ± 0.4 μg/dry weight) and control (0.49 ± 0.03 μg/dry weight), respectively. In control and treated larvae, [Na^+^] decreased, though [K^+^] increased, with increasing exposure time. The shape of the thermal reaction curve of AMP-depended protein kinase and protein phosphatase 2C followed the same norm, which was different from protein phosphatase 1 and protein phosphatase 2A. Protein phosphatase 2A and 2C showed a complete difference in thermal reaction norms. Indeed, thermal fluctuation caused the highest changes in the activity of the enzymes, whereas the RCH showed the lowest changes in the activity of the enzymes. Our results showed a significant enhancement of larval cold tolerance under CA regime, which is related to the high levels of low molecular weight carbohydrates under this regime. Our results showed that among the different thermal regimes tested, the CA larvae had the lowest supercooling point (about -22°C) and the highest cold hardiness following 24 h exposure at -15 and -20°C.

## Introduction

The Khapra beetle, *Trogoderma granarium* Everts (Coleoptera: Dermestidae), is an important and destructive insect pest of stored products. The native distribution of this pest is not known for certain, but this beetle is found in hot dry areas, and it is believed that this pest originated from the Indian subcontinent (Banks, 1977). This pest causes economic losses, particularly in tropical and subtropical regions of Asia and Africa (Burges, 2008). Infestation of seeds and food commodities by *T. granarium* larvae and their cast skins and hairs cause loss of biomass and food quality of stored products. In response to food shortage and unfavorable conditions (i.e., temperatures below 30°C), the larvae enter diapause, remain relatively inactive, and rarely feed. Diapausing larvae tend to leave the food and aggregate in crevices of buildings (Burges, 1962). Based on our previous study, larvae of *T. granarium* are freeze-avoidant or freeze-intolerant. Freeze-avoiding species accumulate polyol cryoprotectants in response to harsh environmental conditions. In these species, polyols permit colligative suppression of supercooling point (SCP) to prevent body freezing (Mohammadzadeh and Izadi, 2018).

To overcome adverse effects of low temperature, several physiological mechanisms have been developed in insects of cold and temperate zones. Three main groups of these mechanisms are: (1) physiological, biochemical, and metabolic alterations [cryoprotectant synthesis and synthesis of antifreeze proteins (AFPs) and/or ice-nucleating agents (INAs)], (2) change in cell function (modification of membranes, regulation of ion-homeostasis, and mobilization of cryoprotectants), and (3) alternation in gene expression (upregulation of stress-related genes) (Overgaard et al., 2007, 2014, 2015; Teets and Denlinger, 2013).

Cold hardiness or cold tolerance is the capacity of an organism to survive long- or short-term exposure to low-temperature levels. This capacity highly depends on developmental stage, genetic potential, season, duration of exposure, and nutritional status of the species. The best known and extensively researched mechanisms of insect cold hardiness are by means of carbohydrate cryoprotectants, antifreeze proteins (AFPs), and ice-nucleating agents (INAs) or ice-nucleating proteins (INPs). All contribute to protective mechanisms that deal with problems of ice formation at subzero temperatures (Storey and Storey, 2012; Sinclair et al., 2015; Andreadis and Athanassiou, 2017). The SCP, the temperature at which freezing of a cell initiates, is experimentally determined by detecting the released latent heat of fusion as body water freezes. Insect cold-tolerance strategies are usually determined on the basis of the SCP (Bemani et al., 2012; Sinclair et al., 2015; Mohammadzadeh and Izadi, 2018). Low molecular weight carbohydrates or sugar alcohols as cryoprotectants play an important role in enhancement of insect’s cold hardiness (Storey and Storey, 2012). Acclimation usually has a trend toward higher levels of cryoprotectants, lower SCP, and subsequently higher survival rates. So, in the cold-acclimated (CA) insects, elevation of cold hardiness may be a function of a decrease in SCP and an increase in cryoprotectants synthesis and accumulation. Activation of the intermediary signal transduction enzymes is a key component of the induction and regulation of insect cold hardiness (Pfister and Storey, 2002a). The cyclic AMP-activated protein kinase (cAMPK) is proving to be a major regulator of catabolic vs. anabolic phase in cells, its actions favoring the former and inhibiting the latter. The AMPK was first discovered as a protein kinase that was allosterically activated by cAMP accumulation under low-energy conditions (e.g., hypoxia) and it is often called the energy sensor or the fuel gauge of the cell (Hardie, 2007; Hue and Rider, 2007). The best-known action of AMPK is phosphorylation and inactivation of acetyl-CoA carboxylase (ACC), which inhibits lipogenesis and promotes fatty acid oxidation under energy-limiting conditions. The AMPK activation also exerts inhibitory control over carbohydrate storage (by inhibiting glycogen synthase) and protein synthesis [by activating the protein kinase that inactivates the ribosomal eukaryotic elongation factor-2 (eEF2)]. A series of recent studies have consistently shown AMPK activation in animals transitioning into hypometabolic states (e.g., frog freeze tolerance, turtle, and fish anaerobiosis, nematode dauer) (Rider et al., 2011). Protein phosphatases are a group of signal transducing enzymes that catalyze phospho-ester bond hydrolysis of phosphorylated proteins resulting in dephosphorylation of cellular phosphoproteins (Barford, 1995). Four major subunits of serine/threonine-specific protein phosphatase are protein phosphatase 1 (PP1), PP2A, PP2B, and PP2C (Cohen, 1989). The critical role of PP1 in the control of glycogen phosphorylase (GP) and eventually low-temperature-triggered activation of glycogen breakdown for polyol synthesis have already been identified, but roles of other protein phosphatases in insect cold hardiness have not been demonstrated so far (Hayakawa, 1985; Pfister and Storey, 2002a).

Electrolyte (e.g., sodium, potassium, and chloride) balance in hemolymph inside and outside of the cell membrane regulates nerve and muscle function and maintains acid-base and water homeostasis. Sodium as the main extracellular cation and potassium as the main intracellular cation are responsible for osmotic pressure gradient between the interior and exterior of a cell membrane.

In our study, we hypothesize that variation in cold tolerance in *T. granarium* larvae acclimated with low temperature arises from variation in the ability to change cAMPK and protein phosphatases activities, maintain ion balance, and increase the concentration of cryoprotectant contents in the cold. We thus predict that if acclimated with low-temperature conditions, cold-tolerant *T. granarium* would: (1) have its enzyme activities vary according to the thermal regimes, (2) maintain [Na^+^] and [K^+^] balance in their hemolymph fluid, and (3) allow changes in enzyme activity and ion balance to increase the concentration of cryoprotectant contents and improve cold tolerance.

## Materials and Methods

### Chemicals

All chemicals used for analysis were purchased from Sigma-Aldrich (St. Louis, MO, United States).

### Insect Rearing

The *T. granarium* population used for the experiments was obtained from cultures that had been originated from stored rice seeds from Karaj (Iran) and maintained for 2 years in the Laboratory of Entomology, Vali-e-Asr University of Rafsanjan, Rafsanjan, Iran. The insects were fed on broken wheat seeds (*Triticum aestivum* L.) under a controlled environmental chamber at 33 ± 1°C with 65 ± 5% RH (by using saturated salt solution) and a photoperiod of 14:10 h (L:D), as described by Nouri-Ganbalani and Borzoui (2017).

### Acclimation Treatments

Beetles were raised from egg to the fourth instar in translucent plastic containers (diameter 15 cm, depth 6 cm) with a hole covered by a 50 mesh net for ventilation, containing broken wheat seeds. *T. granarium* fourth instar larvae were divided into four groups: control, CA, fluctuating-acclimated (FA), and rapid cold-hardened (RCH). For control treatment, 100 individuals were put in translucent plastic containers containing food and kept in standard rearing conditions. For CA treatment (Jakobs et al., 2015; with some modification in temperatures and exposing times), 100 individuals were put in translucent plastic containers containing food, cooled in a programmable refrigerator from rearing conditions to 15°C at a rate of 0.5°C min^-1^ and kept at this temperature at 65 ± 5% RH with a 14:10 h (L:D) light cycle for the 10 days. Thereafter, the temperature was lowered to 5°C at the same rate and the larvae were kept at this temperature at 65 ± 5% RH with a 14:10 h (L:D) light cycle for 10 days. For FA treatment (Bale et al., 2001; with some modification in temperatures and exposing times), one hundred individuals were put in translucent plastic cups containing food, cooled in a programmable refrigerator from rearing conditions as explained in the cycle: 240 min at 5°C followed by 20 min at -10°C followed by 240 min at 5°C followed by 940 min at 33°C, at 65 ± 5% RH with a 14:10 h (L:D) light cycle. This cycle was repeated for 10 consecutive days. For RCH treatment (Wang et al., 2011), the larvae were transferred from their rearing conditions to a programmable refrigerator at 0°C for 4 h. After the treatment period, larvae that survived were used for subsequent experiments. The larvae that were able to walk were counted as alive and larvae that were either not showing any movement in their appendages or were moving, but unable to walk, were counted as dead.

### Enzymes Preparation and Assay

The whole body of acclimated larvae of *T. granarium* was used; it was not feasible to separate out individual tissues. For all enzymes, activities were expressed as Unit per gram wet mass. All assays were repeated five times.

#### AMPK

Individuals were rapidly weighed, chilled, and homogenized 1:10 (w/v), with a few crystals of phenylmethylsulfonyl fluoride (PMSF) added, using a precooled homogenizer (Teflon pestle) in ice-cold potassium phosphate buffer (20 mM; pH 6.8), 2-mercaptoethanol (15 mM), and ethylenediaminetetraacetic acid (EDTA) (2 mM). The homogenates were centrifuged at 13000 *g* for 3 min (5°C). Following centrifugation, the supernatant was pooled and stored on ice for subsequent use. The activity of AMPK was assayed by the procedure of Pfister and Storey (2002a). In brief, ^32^P from ^32^P-ATP was incorporated onto Kemptide (LRRASLG), a synthetic phosphate-accepting peptide, in the presence of 0.1 mM adenosine 30,50-cyclic monophosphate. One unit of AMPK activity is defined as the amount of enzyme required to catalyze the incorporation of 1 nmol ^32^P onto the substrate per minute at 23°C.

#### PP1

Individuals were rapidly weighed, chilled, and homogenized 1:3 (w/v) using a precooled homogenizer (Teflon pestle) in ice-cold buffer A [Tris–HCl (20 mM; pH 7.4), EDTA (2 mM), ethylene glycol-bis(β-aminoethyl ether)-N,N,N′,N′-tetraacetic acid (EGTA) (2 mM), X-mercaptoethanol (15 mM)] containing the protease inhibitors: PMSF (1 mM), tosyl phenylalanyl chloromethyl ketone (TPCK) (0.1 mM), aprotinin (1 mg/ml), and benzamidine (5 mM). The homogenates were centrifuged at 1000 *g* for 3 min (5°C). Following centrifugation, the supernatant was carefully collected and assayed immediately for active PP1. Estimates of PP1 activities at physiological levels of modulating proteins and other factors were done based on assays of concentrated extracts. The PP1 activity was estimated at 23°C by monitoring ^32^P cleavage from ^32^P-labeled phosphorylase (Pfister and Storey, 2002b). One unit of PP1 activity is defined as the amount of enzyme required to releases 1 nmol of phosphate per minute at 23°C.

#### PP2

Individuals were extracted as for PP1 except for a 1:10 (w/v) dilution. The homogenates were centrifuged at 13000 *g* for 20 min (5°C). Following centrifugation, the supernatant was carefully collected and desalted by centrifugation at low speed for 1 min (at room temperature) through 5 ml Sephadex G-25 columns equilibrated in ice-cold Buffer A. The eluant was collected, passed through a second, fresh column, and stored on ice for subsequent use. The activities of PP2A and PP2C were assayed by the procedure of Cowan et al. (2000). PP2A activity was measured as the difference in activity in the presence (blank) versus absence of okadaic acid (2.5 nM). To assess the PP2A activity, the reaction mixture containing peptide RRA(pT)VA (150 mM), EGTA (0.2 mM), X-mercaptoethanol (0.02%), and imidazole (50 mM), pH 7.2, and 10 μl of enzyme extract were incubated for 40 min. The reaction was terminated by adding 50 ml of malachite green dye solution [ammonium molybdate (10%) and malachite green dye (2%), both in HCl (4 N) mixed 1:3 v/v and diluted 2:3 v/v with distilled, deionized water, Tween 20 (0.05%), and Triton-X-100(0.05%)] (Ekman and Jaeger, 1993). Reactions were run in 96-well microplates and the absorbance was read at 595 nm. Appropriate blanks, to which TCA had been added prior to the substrate, were prepared for each treatment. The activity of PP2C was assayed as the same except for the presence of okadaic acid (2.5 nM) and incubation of the reaction mixture for 90 min; PP2C was detected as the difference in activity in the absence versus presence of MgCl_2_ (10 mM) (Cowan et al., 2000).

### Ion Concentration

Ion concentrations were measured in the hemolymph (*n* = 5) as previously described by MacMillan et al. (2015b) with some modification. The [K^+^] and [Na^+^] were measured in the hemolymph at 0, 1, 2, 4, 6, and 12 h after exposure to -10°C in individuals treated with different thermal regimes (100 individuals from each treatment and time point). Hemolymph was sampled from 200 fourth instar larvae (and weighed, to estimate volume), using a micropipette, from an incision made at the coxal joint of a hind leg while applying gentle pressure to the abdomen to allow the hemolymph to flow into the tube. Then, the hemolymph was transferred to a 0.5 ml Eppendorf tube, which was placed in a microcentrifuge (DW-41-230, Radiometer A/S, Brønshøj, Denmark) and spun for 15 s to separate hemolymph from debris. Afterward, 1–5 μl sample of hemolymph was transferred by pipette to a 2 ml buffer solution containing 100 ppm lithium salt. After the preparation was made as described earlier, the [Na^+^] and [K^+^] were measured from the hemolymph using an atomic absorption spectrometry (AAS; Model iCE 3300, Thermo Scientific, Waltham MA, United States) and comparisons to standard curves.

### Whole-Body Glycogen and Sugar Alcohols Quantification

The whole-body glycogen and polyol profiles of acclimated larvae of *T. granarium* were repeated with five replicates (one individual from each treatment and time point) for each experiment at the end of the thermal regimes. All concentrations are expressed as microgram per dry weight.

#### Glycogen

The glycogen content of the larvae was estimated using the modified anthrone method as described by Heydari and Izadi (2014). The larvae were weighed and homogenized in 200 μl of 2% Na_2_SO_4_. Thereafter, 1300 μl chloroform-methanol (1:2) was added to the homogenate. The homogenates were centrifuged for 10 min at 7150 *g* and the supernatant was removed. The pellet was washed in 400 μl of 80% methanol and 250 μl distilled water was added before the heating for 5 min at 70°C. Subsequently, 200 μl of the solution was incubated with 1 ml of anthrone for 10 min at 90°C. After cooling at room temperature, the absorbance of the solution was measured at 630 nm. The glycogen content was determined by comparison to a standard curve that was prepared using glycogen.

#### Sugar Alcohols

The extraction, derivatization, and analytical procedures (gas chromatography coupled to mass spectrometry) were similar to those described by Khani et al. (2007). After the weighing and homogenization of individual larvae in 1.5 ml of 80% ethanol and centrifugation (twice repeated), the supernatant (20 ml) was run along with standards of polyols from 1500 to 5500 ppm. Trehalose, sorbitol, myo-inositol, and glucose were analyzed by HPLC (Knauer, Berlin, Germany) using a carbohydrate column with 4 μm particle size (250 mm × 4.6 mm, I.D., Waters, Ireland) (Khani et al., 2007).

### Cold-Tolerance Assays

In total, two separate experiments were done to study cold tolerance: (1) an acute cold-tolerance assay at subzero temperature for 1 h and (2) an experiment measuring cold-tolerance at -5, -10, -15, and -20 for 24 h. Five replicates and 15 larvae for each replicate were used at each treatment and temperature point. To estimate the acute cold exposure, the larvae treated with different thermal regimes were exposed to acute low temperatures (-5 to -20 °C). Survival rate was assessed after 1 h. Finally, LT80-1 h was calculated as the lowest temperature at which 80% of the larvae died after 1 h exposure (Sinclair and Rajamohan, 2008). To estimate the cold tolerance, the larvae treated with different thermal regimes were kept in a programmable refrigerated test chamber, where temperature was lowered slowly (0.5°C min^-1^) from experimental conditions to the desired treatment temperature (-5, -10, -15 and -20 ± 0.5°C) and held at each temperature for 24 h. The mortality of larvae was recorded via direct observation. The larvae showing no movement in their appendages were judged to be dead (Mohammadzadeh and Izadi, 2016).

### Determination of SCP

The SCP was determined for the acclimated larvae of *T. granarium* (*n* = 15). To determine SCP, individual larvae were placed on a thermocouple (NiCr–Ni probe) connected to an automatic temperature recorder (Testo 177-T4, Testo, Germany) within a programmable refrigerated test chamber. The temperature of the refrigerated test chamber was reduced from experimental conditions to -30°C, at a rate of 0.5°C min^-1^. The lowest temperature reached before an exothermic event that occurred caused by the release of latent heat was taken as the SCP of the individual (Mohammadzadeh and Izadi, 2016).

### Statistical Analysis

Data were initially tested for normality (Kolmogorov–Smirnov test) and homoscedasticity (Levene’s test) before subjecting them to ANOVA. All the data were analyzed using SAS ver.9.2 program (PROC GLM; SAS, 2009). Statistical analyses were performed, based on a completely randomized design, using one-way analysis of variance (ANOVA) followed by a *post hoc* Tukey’s test at α = 0.05.

## Results

### Effect of Thermal Regimes on Enzymes Activity

**FIGURE 1:**
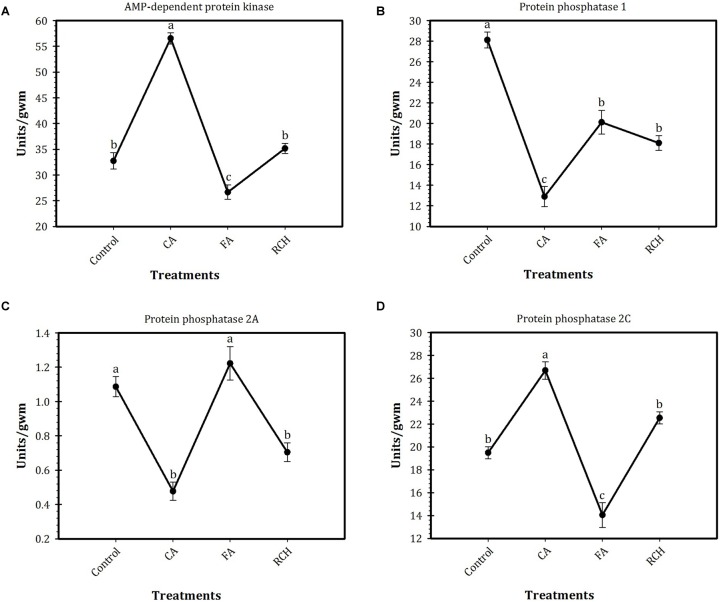
Profiles of AMP-dependent protein kinase (AMPK) and protein phosphatase (PP) activities in *Trogoderma granarium* fourth instar larvae following different thermal regimes. **(A)** total PKA, **(B)** total PP1, **(C)** PP2A, **(D)** PP2C. Each point is an average of five replications. The means followed by different letters are significantly different (Turkey’s test, *P* < 0.05).

Profiles of enzymes activities in *T. granarium* under different thermal regimes are shown in Figure 1. Based on the results of this study, the highest and lowest activities of AMPK (57 and 26 units/gram wet mass, respectively) and PP2C (27 and 14 units/gram wet mass, respectively) were observed at CA and FA treatments, respectively. No significant differences were observed in the activities of these two enzymes between control and RCH. Although the activities of these two enzymes changed in the same norm in different regimes, AMPK was found to be much more active than PP2C. In control, the activity of AMPK was about 33 units/gram wet mass, whereas the activity of PP2C was about 19 units/gram. The activity of both enzymes increased by CA and reached to the highest levels of 55 and 27 units/gram, respectively. The activity of PP1 and PP2A also showed more or less the same norm under different regimes. These norms were completely different from those of the two other enzymes. The highest (28 units/gram wet mass) and lowest (12 units/gram wet mass) activities of PP1 were observed at control and CA treatments, respectively. In the case of PP2A, the highest level of activity was recorded for control and FT treatments (1.1 and 1.2 units/gram wet mass, respectively), whereas the lowest level of activity was shown in CA and RCH (0.5 and 0.6 units/gram, respectively). In PP1, the highest (28 units/gram wet mass) and lowest (13 units/gram wet mass) levels of activity were observed in control and CA regimes, respectively.

### Effects of Thermal Regimes on Hemolymph Na^+^ and K^+^ Concentrations

**FIGURE 2:**
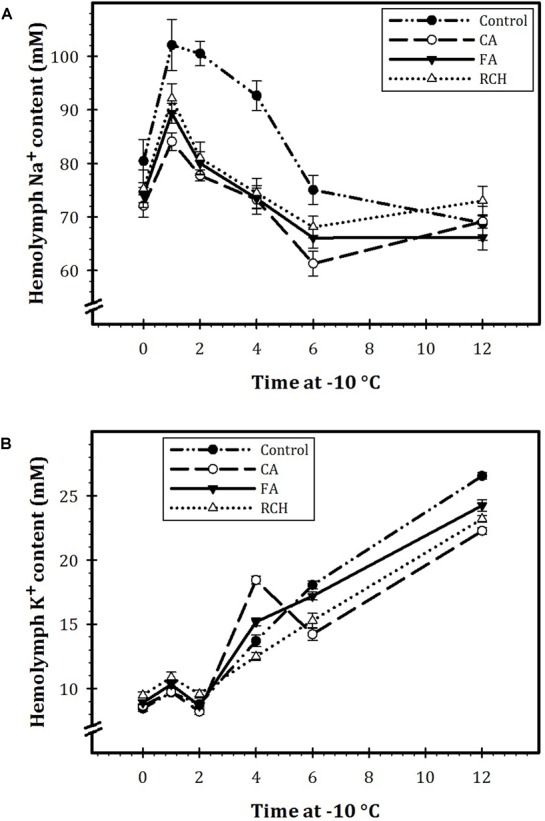
Hemolymph Na^+^ **(A)** and K^+^ **(B)** concentrations before and immediately following 1, 2, 4, 6, and 12 h at –10°C in *Trogoderma granarium* fourth instar larvae following different thermal regimes. Each point is an average of five replications. The means followed by different letters are significantly different (Turkey’s test, *P* < 0.05).

Our results showed that in all the regimes as well as in the control, the concentration of Na^+^ decreased, whereas the concentration of K^+^ increased with an increase in exposure time of the larvae at -10°C (Figure 2). In all the regimes, at the beginning time of exposure (0 h at -10°C), the concentration of Na^+^ was about 70 mM. The concentration of Na^+^ increased and reached to the highest level after 1 h exposure at -10°C. Then, the concentration of this ion in control and the steady state at different thermal regimes decreased with increasing exposure time. The sodium/potassium ratio decreased with an increase in exposure time. During different times of exposure at -10°C, the highest and lowest levels of Na^+^ and K^+^ were recorded for control and CA treatment, respectively. In addition, at the highest levels, the concentration of Na^+^ was about four times more that of K^+^.

### Effects of Thermal Regimes on Carbohydrate Contents

Glycogen content in control larvae with 34.4 mg/dry weight was at the highest level and reached the lowest level of 23.0 in CA larvae. No significant differences were observed between glycogen contents in FT, RCH, and control (Table 1). Trehalose and myo-inositol were found to be the predominant low molecular weight of carbohydrates in all the regimes. Changes in low molecular weight carbohydrate contents were reversely proportional to change in glycogen content. The highest and lowest contents of trehalose and myo-inositol were observed in CA (16.5 mg/dry weight) and control (9.9 mg/dry weight), respectively. No significant differences were observed in sorbitol contents in control at different thermal regimes. The highest and lowest glucose contents were observed in control (0.49 mg/dry weight) and CA (0.14 mg/dry weight), respectively.

**Table 1:** Carbohydrate contents (*n* = 5) of *Trogoderma granarium* fourth instar larvae following different thermal regimes.

Treatments	Carbohydrate contents (μg/dry weight)				
	Glycogen	Trehalose	Sorbitol	Myo-inositol	Glucose
Control	34.4 ± 2.3^a^	9.9 ± 0.7^c^	2.3 ± 0.1^a^	6.0 ± 0.6^c^	0.49 ± 0.03^a^
Cold acclimation	23.0 ± 1.6^b^	16.5 ± 1.0^a^	3.0 ± 0.1^a^	10.7 ± 0.4^a^	0.14 ± 0.02^c^
Fluctuating acclimation	28.1 ± 3.0^ab^	14.2 ± 0.9^ab^	2.8 ± 0.2^a^	10.0 ± 1.0^ab^	0.35 ± 0.01^b^
Rapid cold–hardening	33.5 ± 2.5^a^	12.2 ± 0.6^bc^	2.6 ± 0.2^a^	8.2 ± 0.6^b^	0.37 ± 0.06^ab^
*df*	3, 16	3, 16	3, 16	3, 16	3, 16
*F*	4.86	11.99	2.99	9.07	14.43
*P*	0.0137	0.0002	0.2619	0.0010	<0.0001

*The means followed by different letters in the same columns are significantly different (Turkey’s test, P < 0.05).*

### Effect of Thermal Regimes on SCP and Survival of the Larvae

Data in Table 2 showed that under CA and FT thermal regimes, SCPs of the larvae decreased to the lowest level (about -22°C), which were significantly lower than SCPs of control and RCH regimes. No significant difference was observed between SCPs of control and RCH.

**Table 2:** Relationship between low temperature survival rate (*n* = 5) and supercooling points (*n* = 15) of *Trogoderma granarium* fourth instar larvae following different thermal regimes.

Treatments	SCPs (°C)	Survival rate (%)			
		-5°C/24 h	-10°C/24 h	-15C/24 h	-20°C/24 h
Control	-16.4 ± 0.6^a^	89.3 ± 3.4^a^	54.7 ± 5.3^bc^	9.3 ± 4.0^b^	00.0 ± 0.0^b^
Cold acclimation	-22.7 ± 0.8^b^	96.0 ± 2.7^a^	85.3 ± 3.9^a^	64.0 ± 4.5^a^	20.0 ± 4.7^a^
Fluctuating acclimation	-21.3 ± 0.4^b^	100.0 ± 0.0^a^	69.3 ± 4.5^ab^	48.0 ± 5.7^a^	12.0 ± 4.4^ab^
Rapid cold–hardening	-17.9 ± 0.6^a^	93.3 ± 3.6^a^	41.3 ± 3.9^c^	1.3 ± 1.3^b^	00.0 ± 0.0^b^
*df*	3, 56	3, 16	3, 16	3, 16	3, 16
*F*	23.33	2.52	12.38	44.93	9.19
*P*	<0.0001	0.1949	0.0002	<0.0001	0.0009

*The means followed by different letters in the same columns are significantly different (Turkey’s test, P < 0.05).*

**FIGURE 3:**
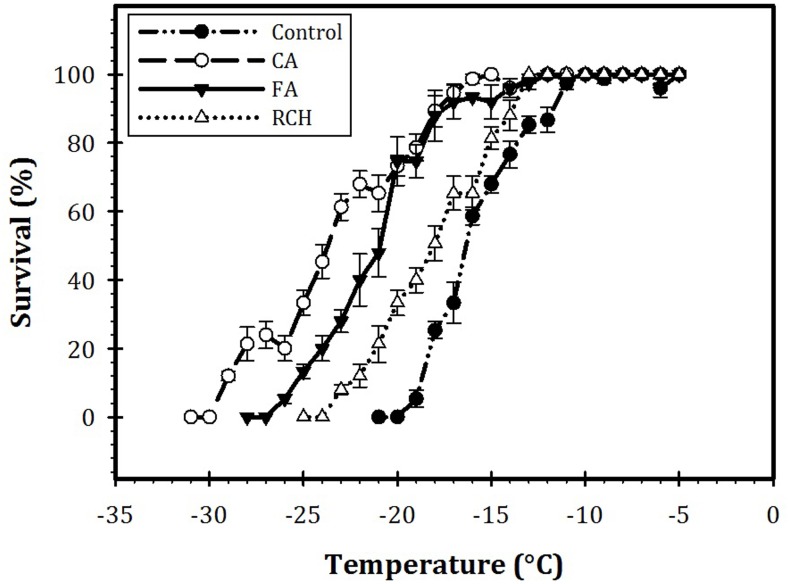
Survival of *Trogoderma granarium* fourth instar larvae following different thermal regimes after acute low-temperature exposure.

Our results also showed a profound effect of CA on the LT_80_ value of the larvae (Figure 3). The LT_80_s of the larvae at control, RCH, FT, and CA regimes were calculated as 11, -14, -19, and -21°C, respectively. In CA larvae, the temperature required for 80% mortality decreased by about 10°C compared with control larvae. In addition, the temperature necessary for the beginning of mortality in the CA regime was 10°C lower than that of control. In all the regimes, the survival rate of the larvae decreased with a decrease in the temperature and increase in the exposure time. CA larvae showed the highest cold hardiness in the -15 and -20°C range. In control, RCH, FT, and CA, larval mortality began at -20, -25, -27, and -30°C, respectively.

## Discussion

In this study, three different thermal regimes (CA, FA, and RCH) were examined. Out of these thermal regimes, substantial effect of CA on physiological adaptations (i.e., cryoprotectant accumulation and enzyme activity) and cold tolerance of the last instar larvae of the Khapra beetle, *T. granarium*, is highly obvious. In addition, results of the current study demonstrated a strong correlation between carbohydrate contents and cold tolerance of the larvae. The CA showed the highest impact on physiological adaptations and subsequently the survival rate of the larvae. In CA larvae, glycogen and SCP were at the lowest levels, whereas low molecular weight carbohydrates (e.g., trehalose), AMPK activity, and survival rates were at the highest levels. The decrease in SCP was proportional to an increase in cold hardiness of the larvae, which, in turn, was associated with an increase in the enzyme activity and cryoprotectant accumulation. Thus, a strong relation between enhanced cold hardiness, elevated enzyme activities, accumulated cryoprotectants, and decreased SCP of the Khapra beetle CA larvae can be concluded from our results. On the other hand, in the CA larvae, enhancement of cold hardiness is a function of both a decrease in SCP and an increase in trehalose synthesis and accumulation. Hiiesaar et al. (2001) showed that the mean SCP of *Leptinotarsa decemlineata* (Say) (Col.: Chrysomelidae) decreased from -10.5 in non-acclimated to -17.5°C in CA adult beetles. In *Hermetia illucens* (L.) (Dip.: Stratiomyidae) prepupae, the SCP was unaffected by cold acclimation, but cold hardiness increased in comparison to control (Spranghers et al., 2017). Insects also have the capability to improve cold tolerance and survival rate in a short period, called rapid cold-hardening (RCH) (Teets and Denlinger, 2013). In the current study, RCH had no significant effects on enzyme activities, survival rates, and cryoprotectant accumulations. This correlates well with the previous studies. Overgaard et al. (2014) in adults of *Drosophila melanogaster* Meigen (Dip.: Drosophilidae) reported no effect of RCH on the activity of GP. They found a small increase in glucose content, whereas, trehalose content remained unchanged following RCH. Kelty and Lee (2001) in RCH adults of *D. melanogaster* found no change in the levels of Hsp70 and carbohydrate cryoprotectants. In disagreement with our results, Lee et al. (2006) reported that RCH significantly increased survival of *Belgica antarctica* Jacobs (Dip.: Chironomidae) larvae. Lee et al. (2006) determined that RCH increased membrane fluidity of fat body cells of *Sarcophaga bullata* (Parker) (Dip.: Sarcophagidae) adult flies. They suggested that “membrane characteristics may be modified very rapidly to protect cells against cold-shock injury”. In adults of *Thrips palmi* Karny (Thysan.: Thripidae), RCH caused accumulation of cryoprotectants mainly trehalose (Park et al., 2014). So, based on our results and results of other researchers, it is reasonable to conclude that insects may become cold hardy by RCH if RCH participates in the accumulation of cryoprotectants (polyols and sugar alcohols). In our study, no accumulation of cryoprotectants and consequently no cold hardiness were determined in RCH regime. Jakobs et al. (2017) found no evidence that acute cold tolerance of *Drosophila suzukii* larvae could be improved by RCH.

The AMPK as a downstream component of a kinase cascade and a key component of energy homeostasis has several functions (regulation of glycogen, sugar, and lipid metabolism) and cellular targets. This enzyme can be regulated by a growing number of hormones (e.g., leptin and adiponectin, insulin, interleukin-6, resistin, TNF-alpha, and ghrelin) and cytokines (Dzamko and Steinberg, 2009; Hardie, 2010; Lee et al., 2010). Our findings showed that AMPK is a predominant signal transduction enzyme of *T. granarium* last instar larvae. Activities of the tested enzymes could be rated as AMPK > PP1 > PP2C > PP2A. The results of some previous studies support the findings of the current study. Pfister and Storey (2006a) demonstrated changes in the activities of AMPK, PP1, PP2A, and PP2C in a freeze-avoiding insect, *Epiblema scudderiana* (Clemens) (Lep.: Olethreutidae) in winter and during exposure of the larvae to subzero temperatures. They demonstrated a limited change and the role for AMPK in overwintering larvae, but the activities of PP2A and PP2C increased when larvae were exposed to -20°C. In another research, Pfister and Storey (2006b) studied changes in the activities of the same enzymes of the goldenrod gall fly, *Eurosta solidaginis* Fitch (Dip.: Tephritidae). They showed increases in AMPK and a decrease in PP1 activity over the winter season and/or at subzero temperature. However, the findings of the present research revealed that the shape of the thermal reaction curve in AMPK and PP2C follows the same norm, which is different from those of PP1 and PP2A. The PP2A and 2C showed opposite trends in activity and different thermal reaction norms. Indeed, thermal fluctuation caused the highest changes in the enzyme activities, whereas larvae of RCH treatment showed the lowest level of the enzyme activities. Decrease in glycogen content and increase in activity of AMPK and cryoprotectant contents in CA larvae of *T. granarium* suggest a role for coarse control of AMPK in the conversion of glycogen reserves into cryoprotectant synthesis and accumulation. Increase in cryoprotectant contents results in enhancement of cold tolerance and survival of the CA larvae. Our previous study has shown that larvae of *T. granarium* are freeze-avoidant or freeze-intolerant (Mohammadzadeh and Izadi, 2018). In these freeze-intolerant larvae, several metabolic adaptations, including synthesis of polyols and low molecular weight carbohydrates (as cryoprotectant), have been developed for survival at subzero temperatures and harsh environmental conditions. The results of the current study indicated a significant enhancement in larval survival and cold tolerance under CA regime. The results of our study also revealed the profound impact of CA on carbohydrate contents of the larvae. In the CA larvae, cold hardiness of the larvae was at the highest value and major cryoprotectants such as trehalose and myo-inositol were at the highest levels, but glycogen reached the lowest concentration. So, it could be concluded from these results that cold acclimation is important in the conversion of glycogen to low molecular weight carbohydrates, which act as a cryoprotectant to enhance cold hardiness of the larvae. High level of cryoprotectants (e.g., trehalose) is essential for reduction of supercooling in freeze-avoidant species or to prevent intracellular ice formation in freeze-tolerant insects. The induction of insect cold hardiness and related adaptations require the intermediary action of signal transduction enzymes (Pfister and Storey, 2006b). In agreement with these aspects, in the current study, the activity of AMPK, as a signal transduction enzyme in the CA larvae, increased and reached the highest level. An increase in the activity of this enzyme was coincident with the increase of cold hardiness, cryoprotectants concentration, and survival rate. These findings strongly support a regulatory role for AMPK and PP1 in the synthesis of cryoprotectants from glycogen. In the CA larvae, glycogen content decreased with the increase in trehalose content and AMPK activity. So, it is reasonable to conclude that AMPK may be responsible for shutting down glycogen synthesis and activating conversion of glycogen to trehalose. For cryoprotectants synthesis, conversion of glycogen to polyols or sugar alcohols is necessary (Storey and Storey, 2012). In this process, the role of PP2C is much more limited than AMPK and there is no role for PP1 and PP2A. In overwintering larvae of *E. scudderiana*, PP1 was found to be responsible for shutting off glycogenolysis, whereas a limited role was attributed to PKA. In this moth, the activity of PP2A and PP2C increased by exposing the larvae to -20°C (Pfister and Storey, 2006a). Some recent studies suggest a big role for AMPK in insect cold hardiness and diapause. Rider et al. (2011) showed a twofold higher activity of AMPK in winter larvae of *E. solidaginis* and *E. scudderiana* in comparison to summer ones. Joanisse and Storey (1995) found an increase in activities of glycogenolytic and hexose monophosphate shunt enzymes in CA *E. scudderiana* larvae, which resulted in the conversion of glycogen into glycerol as a cryoprotectant. In *E. solidaginis* CA larvae, an increase in activity of GP with a decrease in activity of glycolytic enzymes may be responsible for the temperature-dependent switch from glycerol to sorbitol synthesis. In our study, trehalose content was at the highest level in CA larvae. Trehalose, as a cryoprotectant, contributes to stabilizing the lipid bilayer of the cell membrane (Crowe et al., 1992; Storey and Storey, 2012). The results of this study are in agreement with the results of Pfister and Storey (2006b). They showed several metabolic adaptations in freeze-tolerant larvae of the goldenrod gall fly for subzero survival. Several other studies reported this sugar as a cryoprotectant in cold hardy insect species (Behroozi et al., 2012; Bemani et al., 2012; Sadeghi et al., 2012; Heydari and Izadi, 2014; Mohammadzadeh and Izadi, 2016). In agreement with our results, Mohammadzadeh et al. (2017) showed that high cold tolerance of larvae of *Eurytoma plotnikovi* (Hym.: Eurytomidae) was not associated with accumulation of cryoprotectants during overwintering. Enhancement of cold tolerance in insects mostly relies on colligative effects through accumulation of high concentration of cryoprotectants such as trehalose and glycerol to depress SCP (Bemani et al., 2012; Hayward et al., 2014; Heydari and Izadi, 2014). In some insects, if cold exposure induces accumulation of cryoprotectants and subsequently elevates cold hardiness of the insect, SCP remains unchanged. In these insects, cold hardiness enhances through a non-colligative mechanism (Kostal et al., 2001). Cold-induced gene recognition usually consists of two steps: identification of metabolic adaptations that support cold hardiness (e.g., cryoprotectant synthesis) and determination of induced or upregulated proteins/enzymes which support this function (Storey and Storey, 2001).

Chilling insect at low temperature causes a loss of extracellular ions and water homeostasis (MacMillan et al., 2015a). In a recent experiment, MacMillan et al. (2015a) examined the capacity of chill susceptible *Drosophila* species malpighian tubules (MT) and demonstrated that MT lost [Na^+^] and [K^+^] selectivity at low temperatures, which participate in a loss of Na^+^ and water balance and an increase in extracellular [K^+^]. These findings strongly support the results of the current study. Based on our results, exposure of the larvae to low temperature caused a substantial decrease in [Na^+^] and an increase in [K^+^]. This finding strongly supports the concept that low temperature reduced [Na^+^] and [K^+^] selectivity of MT, which contributed to a decrease in [Na^+^], a harmful increase in [K^+^], and consequently, an accumulation in chill injuries. Insect cold hardiness is strongly associated with the ability of MT to retain ions (particularly, K^+^ and Na^+^) and water balance during cold exposure (MacMillan et al., 2015a; Andersen et al., 2017). MacMillan et al. (2015a) concluded that chill-tolerant *Drosophila* species maintained K^+^ secretion better than chill-susceptible species and suppressed K^+^ reabsorption during cold exposure. These data, therefore, are strongly in agreement with our findings. As our results show, at the beginning of the cold exposure, hemolymph [Na^+^] is at the highest level, whereas; [K^+^] is at the lowest level. By increasing exposure time, [Na^+^] decreased and [K^+^] increased. In most insects, hemolymph [Na^+^] is significantly higher than [K^+^]. So, Na^+^ ions tend to leak into the gut lumen while K^+^ ions tend to leak into the hemolymph. At normal temperature, passive ion movements from the MT lumen to the hemolymph and from the hemolymph to the MT lumen are regulated by the energy-demanding proton pump located at the apical membrane MT epithelial cells. At low temperature, [Na^+^] leak away from the hemolymph to the MT lumen and so, its concentration reduces in hemolymph leading to increasing [K^+^] in the hemolymph. Increase in hemolymph [K^+^] causes depolarization of cell resting potential and this depolarization may be a primary reason of cold-induced injury (Kostal et al., 2004; MacMillan et al., 2014, MacMillan et al., 2015; Andersen et al., 2017). We can conclude from our results that the gene expression of ion homeostasis and consequently water balance have altered CA larvae of the Khapra beetle. The same results have been reported by Gerken et al. (2015); MacMillan et al. (2015a), and Des Marteaux (2017).

From a practical viewpoint, control of stored product pests relies mostly on the use of fumigants, e.g., methyl bromide. This pesticide is an ozone-depleting fumigant and its application has been restricted worldwide. Thus, there is an increasing interest to find new alternatives of control methods, including the use of low temperatures (Wilches et al., 2017). Cooling of the seeds and commodities near the SCP of the pests for a specific period of time may be an appropriate method for controlling of the *P. interpunctella* and *E. ceratoniae* (Phillips and Throne, 2010; Hagstrum and Phillips, 2017). Hence, the results of the current study strongly suggest phytosanitary temperature treatments as an alternative for methyl bromide and other fumigant pesticides in the control of stored product pests.

## Conclusion

Our results showed that a significant enhancement of larval cold tolerance under CA regime is related to the elevated level of low molecular weight carbohydrates, e.g., trehalose and protein kinase, and phosphatases activities, and hemolymph ion concentrations. This study provides support for the use of phytosanitary temperature treatments as a potential alternative to fumigant insecticide in the control of this stored product beetle. It also highlights the physiological changes that the insect makes to overcome low temperatures.

## Author Contributions

MM and HI conceived and designed the research, conducted the experiments, contributed the analytical tools and analyzed the data, and wrote the manuscript.

## Conflict of Interest Statement

The authors declare that the research was conducted in the absence of any commercial or financial relationships that could be construed as a potential conflict of interest.
